# Ecological and life history traits predict urban tolerance in North American birds

**DOI:** 10.1098/rsos.260953

**Published:** 2026-10-14

**Authors:** Rachel Elizabeth Fanelli, Paul R. Martin, Frances Bonier

**Affiliations:** ^1^ Department of Zoology and Physiology, University of Wyoming, Laramie, WY, USA; ^2^ Department of Biology, Queen's University, Kingston, Ontario, Canada

**Keywords:** birds, climate variability, environmental tolerance, life history, precipitation variability, temperature variability, urban adaptation, urban tolerance

## Abstract

Urbanization poses a drastic threat to biodiversity, but does not affect all species equally. As such, identifying the life history traits and ecological characteristics that distinguish species that tolerate urban areas from those that do not is a central goal in urban ecology. Additionally, understanding whether shared evolutionary history influences urban tolerance could shed light on its evolutionary origins and reveal the extent to which urban landscapes filter out entire taxonomic clades, homogenizing biodiversity. Here, we assessed the relationship between urban tolerance and eight different life history and ecological traits for 431 North American bird species. We found that birds with higher urban tolerance are longer-lived, smaller-bodied, and more likely to be sedentary or mixed migrants than birds with lower urban tolerance. Birds with higher urban tolerance also have broader environmental tolerance, based on breeding range size and estimates of range-wide variation in precipitation, consistent with their greater likelihood of being habitat generalists. We additionally detected a phylogenetic signal for urban tolerance, with several clades sharing similar urban tolerance measures. Our study identifies several mechanisms that likely underlie species differences in urban tolerance and demonstrates how urban areas can filter bird species based on their life history and ecological traits.

## 1. Introduction

The effects of urbanization can be rapid and far-reaching, influencing patterns of biodiversity across geographic scales [1–4]. As habitats are transformed from non-urban to urban areas, the resources that many species need to survive and reproduce are fragmented and destroyed [2,5]. Urban areas are dominated by impervious surfaces (roads and buildings), are louder, brighter, often warmer (heat island effect) and vary in the size and diversity of green spaces and proximity to water bodies [5–10]. Moreover, climate change can amplify the heat island effect, increase variability in precipitation in urban areas and even cause more extreme weather events like heavy rainfall and drought [11–14], further influencing urban biodiversity [15]. With more than 65% of the world's human population projected to live in urban areas by 2050, urban expansion is only expected to increase [4,16]; therefore, understanding why some species thrive in urban habitats, while others struggle to persist or avoid urban areas altogether, will increase our ability to preserve urban biodiversity [11,16–18].

Species possessing particular combinations of ecological, behavioural and life history traits may be better suited to survive and reproduce in urban areas, while species lacking these traits are filtered out of urban landscapes [19–22]. Identifying the mechanisms that explain this among-species variation in the ability to survive and reproduce in urban areas (i.e. urban tolerance) could therefore be important for predicting and mitigating the impacts of ongoing urbanization and conserving biodiversity. Furthermore, if urban tolerance is shaped by shared evolutionary history, closely related species may be more likely to share pre-existing traits that promote or limit urban tolerance than distantly related species [19,20,23–26]. This could lead to the exclusion of entire taxonomic clades from urban landscapes, further homogenizing urban biodiversity.

Much of the research on traits that enable species to thrive in urban environments has focused on birds, because they are sensitive to environmental change, have wide geographic distributions, occupy diverse ecological niches and offer sufficient data to address questions across broad spatial and taxonomic scales [27–32]. Yet, despite some recurring patterns, evidence of specific traits that predict avian urban tolerance is varied and inconsistent. Species occupying a broad range of environmental conditions (generalists) are often more urban tolerant than species with narrower ranges (specialists), as are species with broader dietary and habitat niches [24,26,33–35]. Larger-bodied and larger-brained species sometimes fare better in urban areas than smaller-bodied and smaller-brained species, potentially reflecting cognitive or morphological traits that underlie urban tolerance [19,24,36,37], although this relationship is not universal [22,23,25,26,34]. A species' life history strategy could also impact its urban tolerance. Species that invest more in reproduction are typically more urban tolerant than those with a lower reproductive investment [19,24,26,36,38], but the opposite pattern has also been observed [22,37,39–41]. Less is known about the relationship between longevity and urban tolerance, but some previous findings indicate a positive relationship with urban tolerance in birds [19,26] (but see [34,38]). The link between avian migration strategy and urban tolerance is less straightforward, but some evidence suggests non-migratory (resident) species are more urban tolerant than migratory species [19,22,36]; whereas others report no relationship [37,39,42], or found the opposite relationship [24,34]. The many inconsistencies across past studies can potentially be explained by differences in the geographic focus and scale of studies, taxonomic breadth of avian species considered and the methods used to quantify urban tolerance [25,34,43].

Fortunately, over the past 25 years, advances in the analysis of large-scale community science datasets provided by platforms like eBird and iNaturalist have transformed our ability to address ecological and conservation questions at local and continental scales [27,30,38,44–48]. For example, the immense volume of fine-scale observation and abundance data provided by eBird has been used by wildlife managers to monitor avian populations, and in urban ecology, has facilitated the creation of several continuous indices quantifying species' urban tolerance [24,26,45,47,49–51]. Researchers have developed continuous indices using fine-scale eBird data to quantify urban tolerance at a continental scale, with some using data from specific cities that meet a given criterion (typically population size) [26], and others using data collected across a species' full range, irrespective of the landscape type (urban or non-urban) or size of the urban area [24,25,49,52]. The latter approach likely offers a more comprehensive assessment of urban tolerance, because it better characterizes a species' tolerance of urban relative to non-urban areas, which is important for understanding how species respond to urban landscapes.

Here, we use previously published quantitative estimates of urban tolerance derived from range-wide relative abundance data [49] along with eight different ecological and life history traits for 431 North American bird species spanning 19 orders and 60 families to determine what traits predict among-species variation in urban tolerance. Additionally, we assess the role of shared evolutionary history by testing for a phylogenetic signal for urban tolerance. This study incorporates complete data on several plausible ecological and life history predictors of urban tolerance across a broad taxonomic scope, and, to the best of our knowledge, is the first to demonstrate a clear association between urban tolerance and variation in range-wide precipitation (precipitation variability). Together, our results provide a comprehensive and generalizable assessment of the causes of among-species variation in urban tolerance, contribute to the growing body of literature in urban ecology, and have important implications for predicting species responses to urbanization and guiding conservation efforts.

## 2. Material and methods

### 2.1. Urban tolerance estimates and breeding range shapefiles

We obtained urban tolerance scores for 431 bird species that breed in the contiguous United States and Canada from a previous study [49,53]. Briefly, Fanelli *et al.* [49] developed several urban tolerance indices for bird species using 2018 eBird Status and Trends (S&T) breeding season relative abundance estimates [44] paired with two distinct urban geospatial datasets [54,55]. For this analysis, we used the continuous multivariate urban tolerance index (−PC1 score from [49]) derived from a principal component analysis that included two top-performing, independent urban tolerance indices. The first evaluated a species' abundance in areas classified as urban relative to non-urban areas within its breeding range, while the second evaluated the night-time light intensity of the areas where a species occurs relative to the unoccupied areas within its range (see metadata in [49] for more information). Fanelli *et al.* [49] report several tests of accuracy of the multivariate urban tolerance index, including their ability to distinguish 24 test species with well-characterized urban tolerance or avoidance and their strong correlation with an independent, semi-quantitative estimate of urban tolerance for a subset of the species. The multivariate urban tolerance index has since been used to assess relationships between population declines and urban tolerance in North American birds, finding urban tolerance does not buffer against species' population declines [56]. This metric has also been used to evaluate the relationship between different life history and nesting traits in coastal birds, and has been compared with two alternative urban tolerance metrics: a binary classification that assigned species as either urban or non-urban and the continuous Urban Association Index developed by Neate-Clegg *et al.* [26,38]. Jennings *et al.* [38] revealed significant correlations among all three metrics, supporting the comparability of our findings with studies using alternative urban tolerance indices.

### 2.2. Life history and ecological traits

We considered traits with a plausible biological relationship with urban tolerance, most of which have been assessed in previous studies, allowing for comparisons. We included estimates of body mass, annual reproductive output and maximum lifespan, because these three traits characterize variation along the spectrum of life history strategies [57–59]. We obtained body mass, average clutch size (number of eggs laid per breeding bout) and average number of breeding bouts per year based on data collected from individuals sampled in the USA or Canada and reported in published sources including the Birds of the World (BOW) database [28,60–62]. We multiplied average clutch size by average number of breeding bouts per year to obtain a measure of annual reproductive output (total number of eggs produced per year). We also included a metric of maximum longevity (years) derived from a published source that reported empirical estimates of longevity for many of the world's birds and modelled maximum longevity for the remaining species [63]. This dataset included direct estimates of maximum longevity for 394 of the species in our analyses and modelled estimates for the remaining 37 species. We included maximum longevity as our lifespan metric because it represents the biological limits of a species and is reflective of underlying physiological mechanisms that might contribute to a species' urban tolerance (e.g. basal metabolic rate, immune function, etc.).

We included estimates of several ecological traits in our analyses known to influence a species' habitat requirements and their ability to tolerate urban habitat [17,24,26]. We extracted breeding biome data primarily from Rosenberg *et al.* [32], who used the Avian Conservation Assessment Database's (ACAD, version 2021) primary breeding habitat metric [31]. For species not reported in Rosenberg *et al.*, we used the ACAD database (version 2021) to classify the species' primary breeding biome following the same criteria as Rosenberg *et al.* (see the supplementary files in [32] for more information). We simplified breeding biomes into seven categories: (i) aridlands, (ii) coasts, (iii) boreal forest, (iv) forest (including eastern, western or forest generalists), (v) grasslands, (vi) wetlands, or (vii) habitat generalist (species that occur in three or more broad ACAD primary breeding habitat categories in similar proportions) [32]. We did not group boreal forest into the broad forest category, because of the greater degree of ecological distinction between boreal forests and other North American forest types. We described species' diets using data from the EltonTraits dataset (version 1.0), which provides species-level foraging attributes for birds and mammals [27]. We categorized species as: (i) frugivore/nectivore, (ii) herbivore/granivore, (iii) invertebrate diet, (iv) carnivore (including piscivores and scavengers), or (v) omnivore (when no single diet category comprised 60% or more of a species' diet) [27]. This approach does not directly account for differences in diet during offspring development, when many species adopt an insectivorous diet, despite reliance on other foods during adulthood (e.g. many granivores, frugivores and nectivores). We classified species' migration strategies as: (i) migratory, (ii) mixed strategy (with some populations that do migrate, others that do not and/or populations of irruptive migrants), or (iii) non-migratory (i.e. sedentary) based on descriptions in the BOW species accounts [28].

We included two proxies of environmental breadth in our analyses: geographic range size and two metrics of climatic variability. Species with larger geographic ranges are more likely to experience a wider range of environmental conditions, and some evidence indicates they can be more urban tolerant than species with smaller ranges [24,25,33]; although the relationship between range size and urban tolerance can be influenced by migration status [25]. We calculated breeding range size for each species from the eBird S&T breeding range shapefiles that were also used to calculate species' urban tolerance scores [49]. We measured the area (km^2^) of the breeding range shapefile using the ‘Geometry’ function in ArcGIS (version 10.7.1) [64].

We quantified variation in climatic conditions experienced across a species' breeding range using publicly accessible climate data. Specifically, we collected mean monthly temperature (°C) and precipitation (mm) data at a 0.5° latitude by 0.5° longitude gridded resolution using Climatic Research Unit gridded Time Series data from 1965–2018 (CRUTS v4) [65]. To obtain temperature and precipitation shapefile data specific to areas within a species' breeding range, we clipped the temperature and precipitation shapefiles to each species' eBird S&T breeding range shapefile (or year-round shapefiles for non-migratory species). The resulting shapefile contained mean monthly temperature and precipitation estimates for all grid cells within a species' breeding range while maintaining a 0.5° latitude by 0.5° longitude resolution. We only considered climatic variation during the breeding season, to parallel the approach used to estimate urban tolerance (breeding season relative abundance), so we established each species' breeding months using the breeding season start and end date variables from Fanelli *et al.*, which are linked to species' eBird S&T seasonal relative abundance estimates [29,49]. For the current study, we measure breeding season as the span of months during which breeding occurred in over half of each month. Species that occur in the same location year-round do not have eBird S&T breeding season start and end data, so we established breeding season timing from descriptions of phenology and annual cycle data in the BOW species accounts [28]. We then calculated the standard deviation of mean monthly temperature and mean monthly precipitation across each species' breeding range using climatic data restricted to their species-specific breeding months to obtain two metrics of climatic variability: temperature variability and precipitation variability.

### 2.3. Statistical analyses

We used R version 4.5.2 for all statistical analyses and plotting [66]. We assessed the distribution of all continuous variables and transformed non-normally distributed data using one of the most effective transformations identified using the ‘*bestNormalize’* function in the R package of the same name (version 1.9.2) [67]. We square-root transformed the response variable, urban tolerance (−PC1), adding a constant (+2.05) to make all values positive before transformation. For the predictor variables, we log_10_ transformed body mass (g) and longevity (years), natural log transformed annual reproductive output (number of eggs produced per year), and square-root transformed breeding range size (area) and both climate variability metrics (standard deviation of temperature and precipitation). We scaled all continuous predictor variables after transformation using a *Z*-scale transformation (mean = 0, s.d. = 1) to facilitate comparison of effect sizes. Some of these six continuous predictors were moderately correlated with each other, but collinearity was not an issue (highest correlation was body mass with longevity: Spearman's correlation coefficient = 0.62, all others: *ρ* ≤ 0.35; supplementary material, figure S1).

To identify the ecological and life history traits that predict urban tolerance, we created a global linear mixed effects model (LME) using the ‘*lme*’ function in the package ‘nlme’ (version 3.1-168) [68], with urban tolerance as the response variable and body mass, annual reproductive output, maximum longevity, breeding range size, temperature variability, precipitation variability, breeding biome, migration strategy and primary diet as fixed effects. Additionally, we included taxonomic family as a random intercept in all models as a coarse control for phylogenetic non-independence. We did not include interactions to ensure our global model did not exceed the statistical power of our sample size (i.e. a minimum of 10 observations per estimated parameter [69]) and to better facilitate comparison of our findings with existing literature. We then used model selection to compare all possible recombinant models, including a null model using the ‘*dredge*’ function in the package ‘MuMIn’ (version 1.48.4) [70]. Upon finding significant patterns associated with categorical fixed effects with more than two levels, we conducted post-hoc pairwise contrasts using an estimated marginal means comparison with a Tukey adjustment using the ‘*emmeans*’ function in the R package of the same name (version 2.0.1) [71]. We evaluated fit of the global and top-ranked models by assessing plots of fitted values across standardized residuals, standardized residuals across quantiles of standardized normals, model residuals across each fixed effect and a histogram of model residuals. We performed Bartlett's test to assess homogeneity of variances across categorical factor levels and assessed Cook's distance to identify any overly influential points (all Cook's values were less than 0.05). Upon finding evidence of unequal variance across breeding biome categories in the global model, we added a term to model the variance structure across the levels of this fixed effect, which improved model fit.

We conducted complementary analyses to determine (i) if phylogenetic non-independence influenced the relationships between urban tolerance and ecological and life history traits identified in our main analyses, and (ii) if evolutionary history predicts estimates of urban tolerance. We obtained a phylogeny for our focal species from https://birdtree.org/ [72] by downloading 1000 phylogenetic trees from the ‘Hackett all species' tree set, and then created a maximum clade credibility tree with median node heights based on those 1000 trees using TreeAnnotator in the program BEAST (v.1.8.1) [73]. We imported our final tree into R using the package ‘ape’ (version 5.8.1) [74], then used this phylogeny for the two aims described above.

We repeated the top-ranking model identified in the analyses described above using a Bayesian phylogenetic mixed-effects model with the species phylogeny included as a random effect to assess the potential influence of phylogenetic non-independence using the function ‘*MCMCglmm*’ in the R package of the same name (version 2.36). We specified uninformative priors where V = 1 and nu = 0.002 for both R and G structures [75]. We ran simulations for 200 000 iterations with a burn-in of 50 000 iterations and a thinning interval of 50. We reran each model three times and evaluated convergence between all three runs using Gelman's convergence diagnostic, where the upper limits should approach 1. We also examined trace plots to ensure no trends existed across runs, effective sample sizes were greater than 200, Geweke's convergence diagnostics showed absolute values less than 1.96, and density plots of the posterior distributions showed symmetry and normality [75,76].

We tested for a phylogenetic signal in urban tolerance using the function ‘*phylosignal*’ in the R package ‘phyloSignal’ (version 1.3.1) [77] and set the total number of iterations for estimating *p*-values to 100 000. We calculated five indices that test for a phylogenetic signal using either the autocorrelation principle (Abouheif's Cmean, Moran's I) or an evolutionary model (Blomberg's K, Blomberg's K* and Pagel's Lambda). We then generated a phylogenetic correlogram using Moran's I to assess autocorrelation at different phylogenetic distances (patristic distance) and identified specific regions in the phylogeny where a phylogenetic signal was strongest with a Local Indicator of Phylogenetic Association (LIPA) test using local Moran's I estimates calculated from the patristic matrix.

## 3. Results

Throughout the results, we report linear mixed-effect model effect estimates ± standard error (SE) for continuous predictors, all of which were transformed and scaled before analysis to facilitate comparison. Estimated marginal means ± SE are reported for categorical variables. We report all the top models from the model comparison in the supplementary material, table S1. The full summary of the top-ranked linear mixed-effects model is reported in the supplementary material, table S2, and the pairwise post hoc contrasts for all categorical variables retained in the top model in the supplementary material, table S3.

### 3.1. Predictors of urban tolerance

The top-ranked model retained the fixed effects of body mass, maximum longevity, breeding range size, precipitation variability, breeding biome and migration strategy (supplementary material, table S1). Two other models were ranked within 2 AICc of the top-ranked model. The second-ranked model included all the fixed effects retained in the top model as well as primary diet, while the third-ranked model included all the fixed effects that were in the top model except breeding range area. None of the top models retained annual reproductive output or temperature variability. Because of this consistent support for the importance of almost all the factors retained in the top-ranked model, we report results of that model below.

Smaller-bodied species had higher urban tolerance scores than larger-bodied species (LME, body mass: *ß* = −0.118 ± 0.035, *p* < 0.001; figure 1*a*). Longer-lived species had higher urban tolerance scores than shorter-lived species (LME, maximum longevity: *ß* = 0.090 ± 0.028, *p* < 0.001; figure 1*b*). Species with larger breeding ranges (area) had higher urban tolerance than species with smaller breeding ranges (LME, breeding range size: *ß* = 0.054 ± 0.025, *p* = 0.030; figure 1*c*), and species with breeding ranges with greater variation in precipitation had higher urban tolerance scores than species with breeding ranges with less variable precipitation (LME, breeding range size: *ß* = 0.107 ± 0.024, *p* < 0.001; figure 1*d*). Non-migratory and mixed-migratory species had higher urban tolerance scores (emmeans = 1.50 ± 0.06 SE and 1.36 ± 0.04 SE, respectively) than migratory species (emmeans = 1.21 ± 0.04 SE; figure 2*a*). Habitat generalists had the highest urban tolerance scores (emmeans = 1.67 ± 0.06), while species that primarily breed in boreal forest and grasslands had the lowest urban tolerance (emmeans = 1.01 ± 0.06 SE and 1.13 ± 0.07 SE, respectively; figure 2*b*). Forest species had intermediate urban tolerance scores (emmeans = 1.38 ± 0.04 SE) that were distinct from both the lowest- and highest tolerance groups (i.e. boreal and grasslands versus habitat generalists), whereas coastal, aridland and wetland species also had intermediate urban tolerance scores, but with less clear separation from both extremes (emmeans = 1.34 ± 0.14 SE, 1.48 ± 0.07 SE and 1.48 ± 0.07 SE, respectively; figure 2*b*).

**Figure 1.**
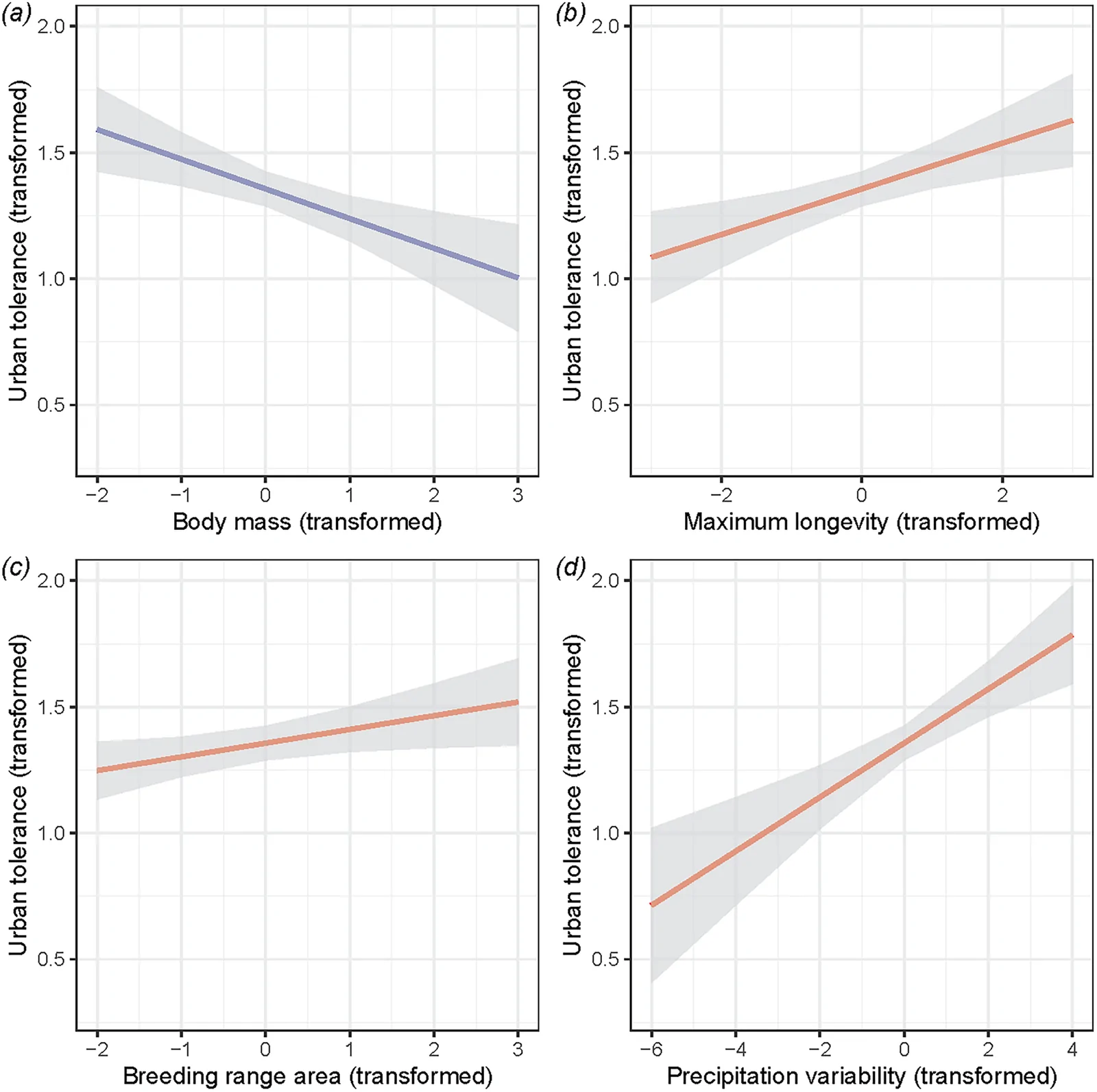
Body mass, maximum longevity, breeding range size and precipitation variability predict variation in urban tolerance in birds (*N* = 431 species). Species with high urban tolerance are smaller (*a*), live longer (*b*), have larger breeding ranges (*c*) and experience greater range-wide precipitation variability (*d*) compared with species with lower urban tolerance. Plots illustrate model-predicted relationships (solid lines) between a species' urban tolerance (square-root transformed, y-axis) and A: body mass (log_10_ transformed and scaled), B: maximum longevity (log_10_ transformed and scaled), C: breeding range size (square-root transformed and scaled), and D: precipitation variability (square-root transformed and scaled), with grey shaded regions representing 95% confidence intervals, and solid lines representing model-predicted values (estimated marginal means across categorical predictor levels, with other continuous predictors held at their mean). The type of relationship is denoted by colour: red = positive, blue = negative. See main text and the supplementary material, tables S2 and S3, for full details.

**Figure 2.**
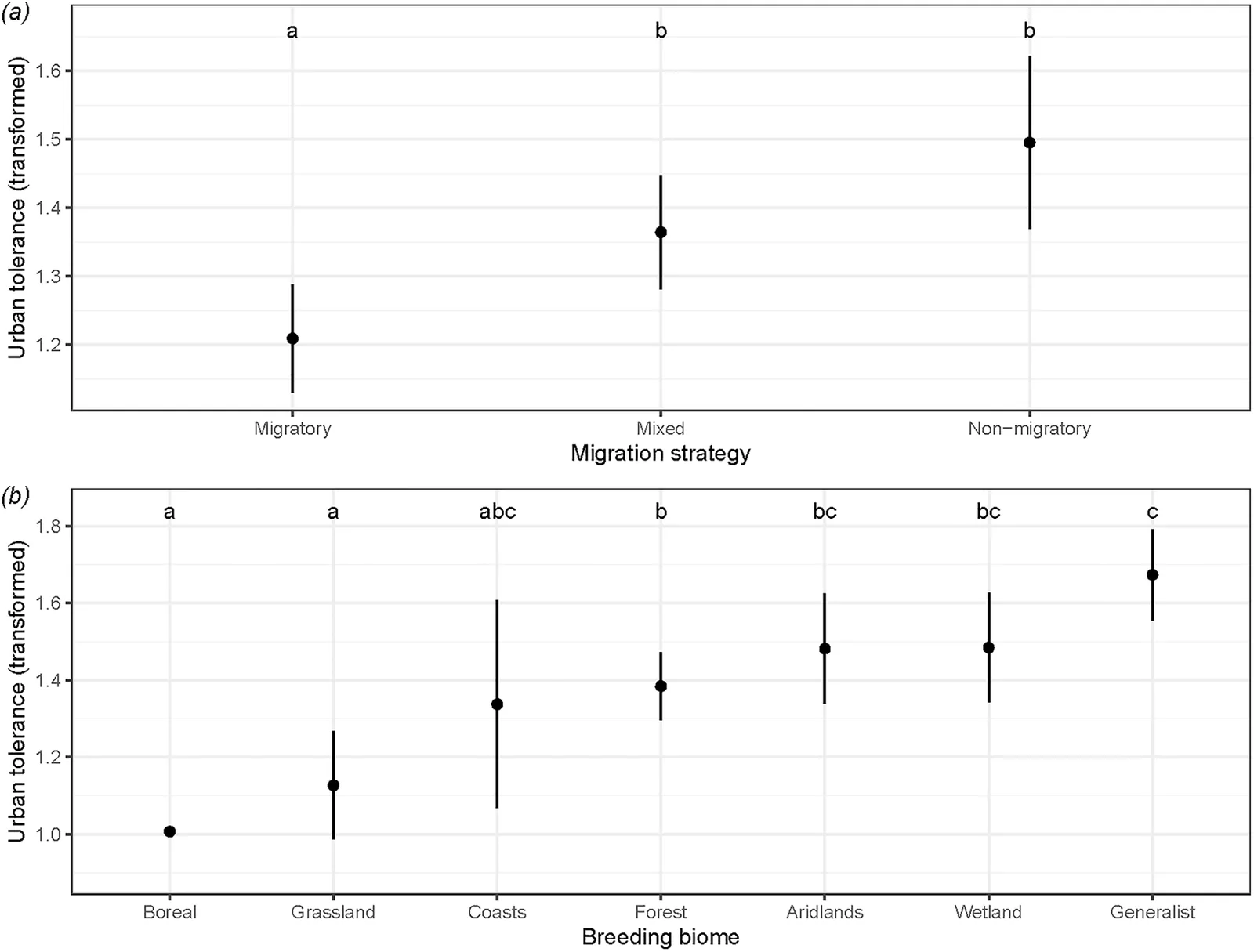
Migration strategy (*a*) and breeding biome (*b*) predict variation in urban tolerance in birds (*N* = 431 species). Points illustrate estimated marginal means and error bars show 95% confidence limits. Estimated marginal means sharing a letter are not significantly different (*p* > 0.05; Tukey adjustment). All plots illustrate relationships controlling for all other effects retained in the model. See main text and the supplementary material, tables S2 and S3, for full details.

### 3.2. Complementary analysis with control for phylogeny

The results of the Bayesian phylogenetic mixed model assessing the potential influence of phylogenetic non-independence produced similar results to the main, non-phylogenetic model. Estimates for all fixed effects (body mass, maximum longevity, breeding range size, precipitation variability, breeding biome and migration strategy) were largely consistent in terms of both direction and magnitude when phylogeny was added as a random effect to the top-ranked model (supplementary material, figures S2 and S3, and tables S4 and S5). However, in the main non-phylogenetic model, mixed-migratory and non-migratory species had higher urban tolerance scores than non-migratory species but did not differ from one another (figure 2*a*; supplementary material, table S3), whereas in the phylogenetic model all three groups differed from one another (supplementary material, figure S3a and table S5). This indicates that phylogenetic non-independence did not bias our main results.

**Figure 3.**
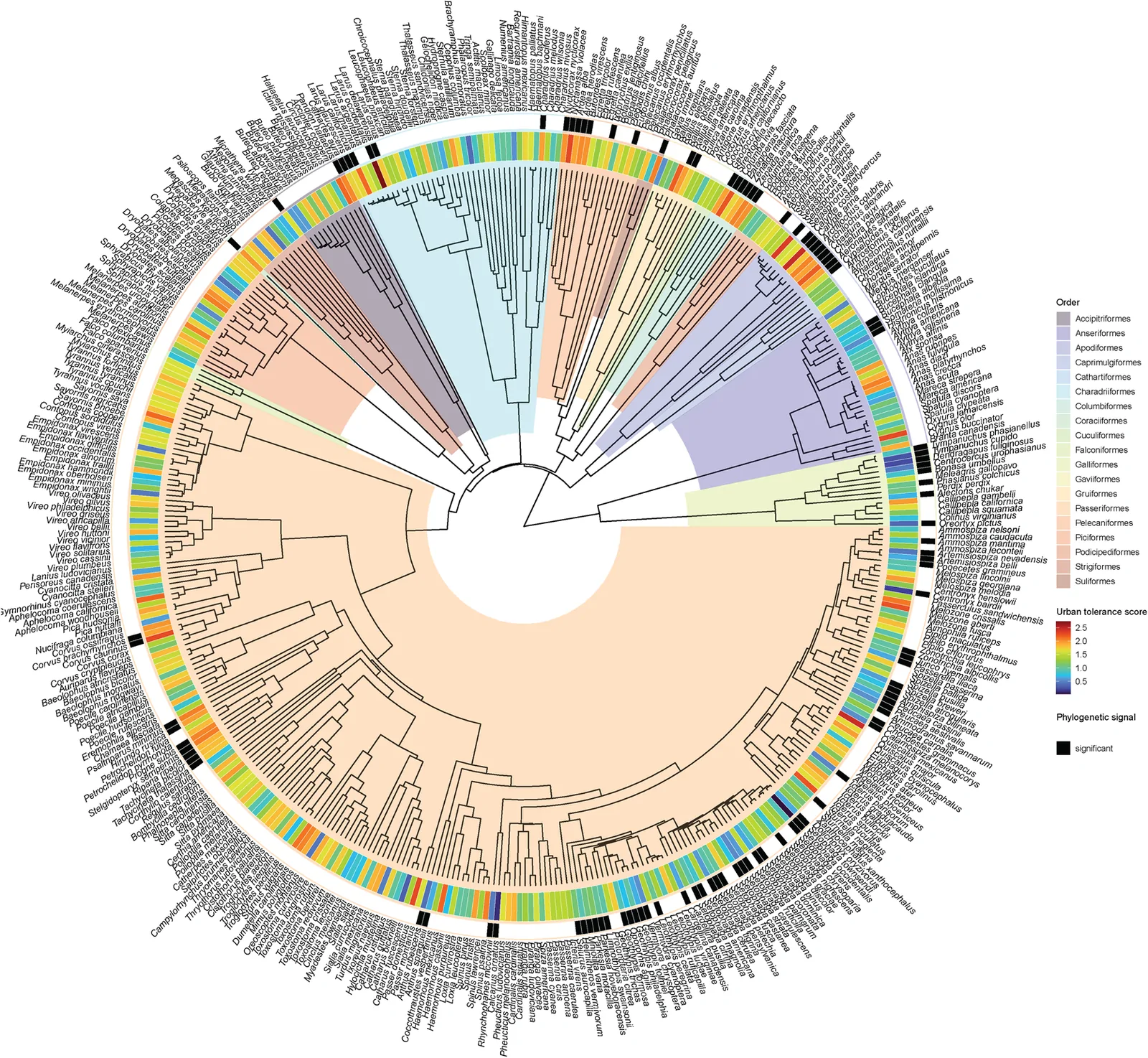
Phylogenetic relationships among 431 North American bird species explain some variation in urban tolerance. Higher values indicate a greater tolerance of urban habitats (red), and lower values indicate a lower tolerance of urban habitats (blue). Black bars next to a species' names indicate evidence of a phylogenetic signal that was detected using a Local Indicator of Phylogenetic Association (LIPA) test of Moran's I estimates. Taxonomic orders are highlighted and distinguished by colour.

### 3.3. Tests for phylogenetic signal in urban tolerance

We detected evidence of a phylogenetic signal for urban tolerance with all five indices that we calculated (Abouheif's Cmean = 0.262, *p* < 0.001; Moran's I = 0.050, *p* < 0.001; Pagel's Lambda = 0.368, *p* < 0.001; Blomberg's *K* = 0.070, *p* < 0.001; and Blomberg's K* = 0.083, *p* < 0.001). Using Moran's I estimates, we found a positive autocorrelation in urban tolerance estimates towards the tips of the phylogenetic tree, in younger, more recently diverged lineages, but no significant autocorrelation among deeper lineages (supplementary material, figure S4). The LIPA test revealed the phylogenetic signal (*p* < 0.05) was explained by 24% (104/431) of the species included in our analyses. Species with both high and low urban tolerance contributed to the phylogenetic signal (figure 3).

## 4. Discussion

We assessed ecological and life history traits that predict differences among 431 North American bird species in their abilities to cope with urban breeding habitats using a quantitative estimate of species-level urban tolerance [49]. We found that urban-tolerant birds are longer-lived and smaller-bodied, and that non-migratory and mixed-migratory species have higher urban tolerance than migratory species. Species with larger breeding ranges and greater range-wide variability in precipitation also have higher urban tolerance than those with smaller breeding ranges and lower precipitation variability, suggesting broad environmental tolerance confers higher urban tolerance. The observation that habitat generalists have the highest urban tolerance further supports this conclusion. We did not find evidence of a connection between urban tolerance and either temperature variability or annual reproductive output. These results were robust, being similar in supplementary analyses that controlled for phylogenetic non-independence, suggesting that differences between our study and others could reflect differences in the taxonomic scope of species included and the spatial scale at which urban tolerance was measured.

We considered several life history traits in our analyses (body mass, maximum longevity and annual reproductive output), finding evidence that both body mass and longevity predicted urban tolerance. In vertebrates, larger animals live longer than smaller animals [78], so the positive relationship between body size and longevity was anticipated (Spearman's correlation coefficient (*ρ*) = 0.62, *p* ≤ 0.001; supplementary material, figure S1). If a particular set of life history traits were favoured in urban environments, we might predict that body mass and longevity would be similarly related to urban tolerance, and yet, we found opposing patterns when both were included in the same models—smaller birds and longer-lived birds were more urban tolerant than larger and shorter-lived birds. Although the relationship between urban tolerance and these two traits might seem contradictory, a similar pattern has been observed in birds at a global scale [26]. These results could indicate that small size and traits associated with it might be more advantageous in urban environments than having traits associated with a slow life history [41]. Small size is associated with reduced resource needs for growth and reproduction [79], which could be advantageous in urban environments where resources might be lower quality than in non-urban environments [5]. Additionally, some urban areas may contain highly fragmented habitat patches too small to support the territory sizes required by larger-bodied species [17,80,81]. By contrast, some evidence suggests that selection favours slower life histories in urban areas, with adult mortality being lower and offspring mortality being higher in some urban populations, relative to non-urban conspecific populations [41]. As such, species that live longer and invest more in parental care of fewer, higher quality young could be at an advantage compared to species with faster life histories, independent of their body size [82]. We would also expect to see smaller species invest more in annual reproductive output as a form of bet-hedging [37], which we did (Spearman's correlation coefficient (*ρ*) = −0.08, *p* ≤ 0.001); however, we did not find evidence of a relationship between urban tolerance and reproductive output. Although we did not find a relationship between reproductive output and urban tolerance, this does not rule out a role for breeding-related traits more broadly; rather, it suggests that other species-specific breeding traits, such as nest-site characteristics, may better predict urban tolerance than reproductive output alone. For example, species that are more flexible in their nesting habitat requirements and/or that breed higher off the ground could be more likely to do better in urban environments as a result [24,26,38], potentially due to increased nest predation and/or disturbance [34,40].

As with previous research, we found migration strategy predicted urban tolerance [25]. Sedentary birds and those with a mixed strategy had the highest urban tolerance, while migratory species had the lowest. Migration is a costly life history strategy, often requiring enormous amounts of resources for long-distance movements, higher risks of mortality and the requirement of repeated location and establishment of home ranges as an individual moves from breeding to non-breeding habitats [83]. In contrast, sedentary species should generally only need to establish a home range once, and might be at an advantage in responding with adaptive plastic behavioural or physiological adjustments to local environments, including urban habitat [84]. Additionally, sedentary species are frequently dominant to closely related migratory species [85], and urban-tolerant species are also often competitively dominant [86]. These two phenomena might be functionally mediated by shared mechanisms such as greater investment in competitive traits in both sedentary and urban birds. Sedentary species may have higher urban tolerance because residency is only viable when local resources are sufficient to meet year-round energetic demands, which is something that urban environments can provide in the form of anthropogenic food subsidies and modified resource landscapes [5,17,22,40,87].

Mounting evidence indicates that urban-tolerant species are often generalists with broad environmental tolerance [24,26], consistent with our findings of increased urban tolerance in species that breed in multiple biomes (i.e. habitat generalists). These species likely encountered a wider range of ecological conditions and selective pressures throughout their evolutionary histories, predisposing them to thrive across a range of environments, with some of these traits fortuitously increasing their fitness in urban areas. In contrast, breeding populations of boreal and grassland birds have experienced a dramatic decline in North America over the past half century [32]. Consistent with this pattern, these species have the lowest urban tolerance scores (figure 2*b*), suggesting continued urban expansion is likely to further reduce their populations, alongside other known drivers of boreal and grassland species decline (e.g. habitat loss and degradation related to agriculture, logging and climate change [32,88–91]).

Species that primarily breed in forest biomes had intermediate urban tolerance scores with respect to the lowest- and highest-scoring biome groups (figure 2*b*), which could reflect some forest species doing well in urban areas and others doing poorly. However, this distinction could also partly reflect the larger sample size in the forest biome category (*n* = 164) relative to the other biome categories, which increased the precision of this estimate and statistical power to detect differences across comparisons (supplementary material, table S3). The forest breeding biome is comprised of species that breed in three different forest habitats: eastern forests, western forests and forest generalists, as we collapsed those three into a single ‘forest’ category to facilitate statistical analyses, at the expense of masking finer-scale variation in urban tolerance among eastern, western and generalist forest species. By contrast, the other categories each had fewer species, and the grassland, coastal, aridland and wetland categories all had wider confidence intervals, with overlap into one of the extreme ends (coastal overlapping both), even after accounting for unequal variance across biome categories, potentially reflecting true within-group heterogeneity, limited statistical power due to sample size or both (supplementary material, table S3).

Broad environmental tolerance that confers higher urban tolerance might not only be common in habitat generalists, but could also be found in species with larger breeding ranges that encounter a variety of climatic conditions. Indeed, we found that species with larger breeding ranges had higher urban tolerance than species with smaller breeding ranges. Although temperature variability and precipitation variability were weakly correlated (Spearman's correlation coefficient (*ρ*) = 0.17, *p* ≤ 0.001), our direct measure of precipitation variability was a stronger predictor of urban tolerance than temperature variability, which was not related to urban tolerance. To the best of our knowledge, our study is the first to demonstrate a clear link between precipitation variability and urban tolerance. Precipitation can influence birds both directly and indirectly, with these effects often especially pronounced during the breeding season [92]. Precipitation can be a strong determinant of local resource availability (both food and water), and so birds that are able to tolerate variable precipitation might have evolved more flexibility or efficiency in resource use to cope with dynamic precipitation. Such flexibility or efficiency could also be beneficial in cities, which are often characterized by stable but lower quality food availability [40].

We found clear evidence of a phylogenetic signal for urban tolerance, indicating that closely related species are more likely to share similar urban tolerances than distantly related species. We detected a phylogenetic signal for both low and high levels of urban tolerance, primarily in more recent lineages, suggesting that the traits that confer high urban tolerance are not highly conserved across deeper lineages. The presence of a phylogenetic signal for urban tolerance also points to the importance of pre-existing traits in facilitating species' tolerance of urban environments. These results add to a growing body of evidence suggesting shared evolutionary history is a strong predictor of urban tolerance, at least in species living in more recently urbanized countries [20,24–26]. However, phylogenetic signal explained urban tolerance in only 24% of species (LIPA test, figure 3), suggesting that while shared evolutionary history predisposes some species to urban tolerance, the majority of variation among species in urban tolerance is likely driven by other factors, including plasticity and/or evolutionary lability related to the ecological and life history traits examined in our analysis.

## 5. Conclusions

Urbanization presents both threats and opportunities for birds, with some species thriving in urban landscapes while others decline [5,10,18,87]. Here, we provide a comprehensive analysis of the traits that distinguish species that thrive in urban landscapes from the many others that do not, using a quantitative urban tolerance metric derived from species' range-wide abundance data. Our findings provide robust insights into the ecological and life history traits that could be key to a species' tolerance of urban environments, which is vital given ongoing urban expansion [4,16]. We found relatively small and long-lived, sedentary and mixed-migratory species with broad environmental tolerance (i.e. habitat generalists that encounter variable precipitation across their large breeding ranges) appear more likely to do well in urban landscapes. Urban biodiversity may therefore be best preserved by targeting urban design and land management efforts towards the ecological needs of these species. Increasing urban biodiversity requires not only that we design urban areas that can attract biota, but also that urban areas can support those organisms' ecological needs, rather than acting as ecological traps. Encouraging species with low and moderate urban tolerance to use urban landscapes would require ensuring that these species have access to the resources that they need during the breeding season. This could include incorporating more heterogeneous green spaces that can support the habitat requirements of larger-bodied birds, habitat specialists and migrants. Maintaining adequate water- and tree-cover in urban areas can also positively impact urban biodiversity and therefore should be a key focus for wildlife conservation managers and urban planners [10].

## Supplementary Material



## Data Availability

Supplementary material is available online [93], and all datafiles associated with this manuscript are stored on Figshare [94].
