# Supplementary material for: Ecological and life history traits predict urban tolerance in North American birds
Source: R Soc Open Sci. 2026 Oct 14;13(10):260953. doi: 10.1098/rsos.260953 (PMC13679050; doi:10.1098/rsos.260953)
Supplement: Supplementary file 1 [file rsos.260953.zip › rsos.260953/rsos260953_si_001.pdf]

Electronic Supplementary Material 1

Ecological and life history traits predict urban tolerance

in North American birds

R.E. Fanelli, P.R. Martin, and F. Bonier

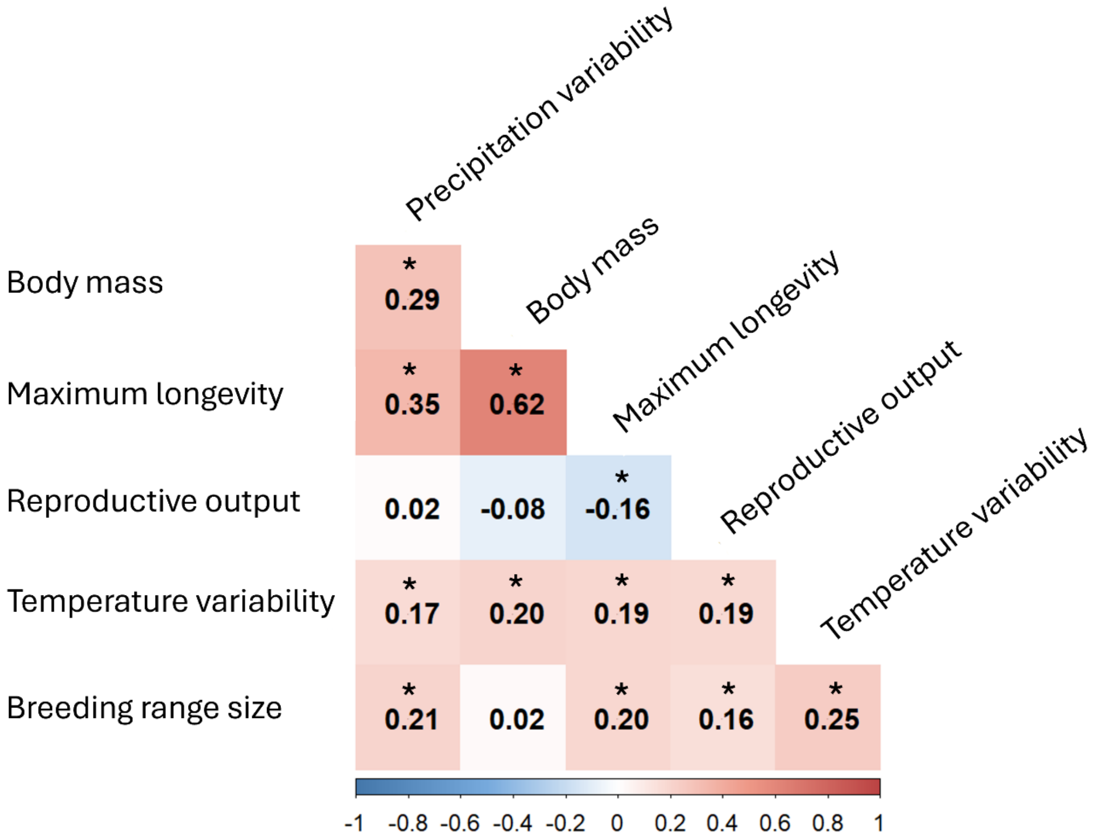

Figure S1. Correlation matrix showing pairwise relationships among ecological, climatic, and life history traits for 431 North American bird species. Values are Spearman's correlation coefficients (ρ), with statistically significant pairwise relationships ($p < 0.01$) denoted by an asterisk (*), positive relationships shaded red, and negative relationships shaded blue.

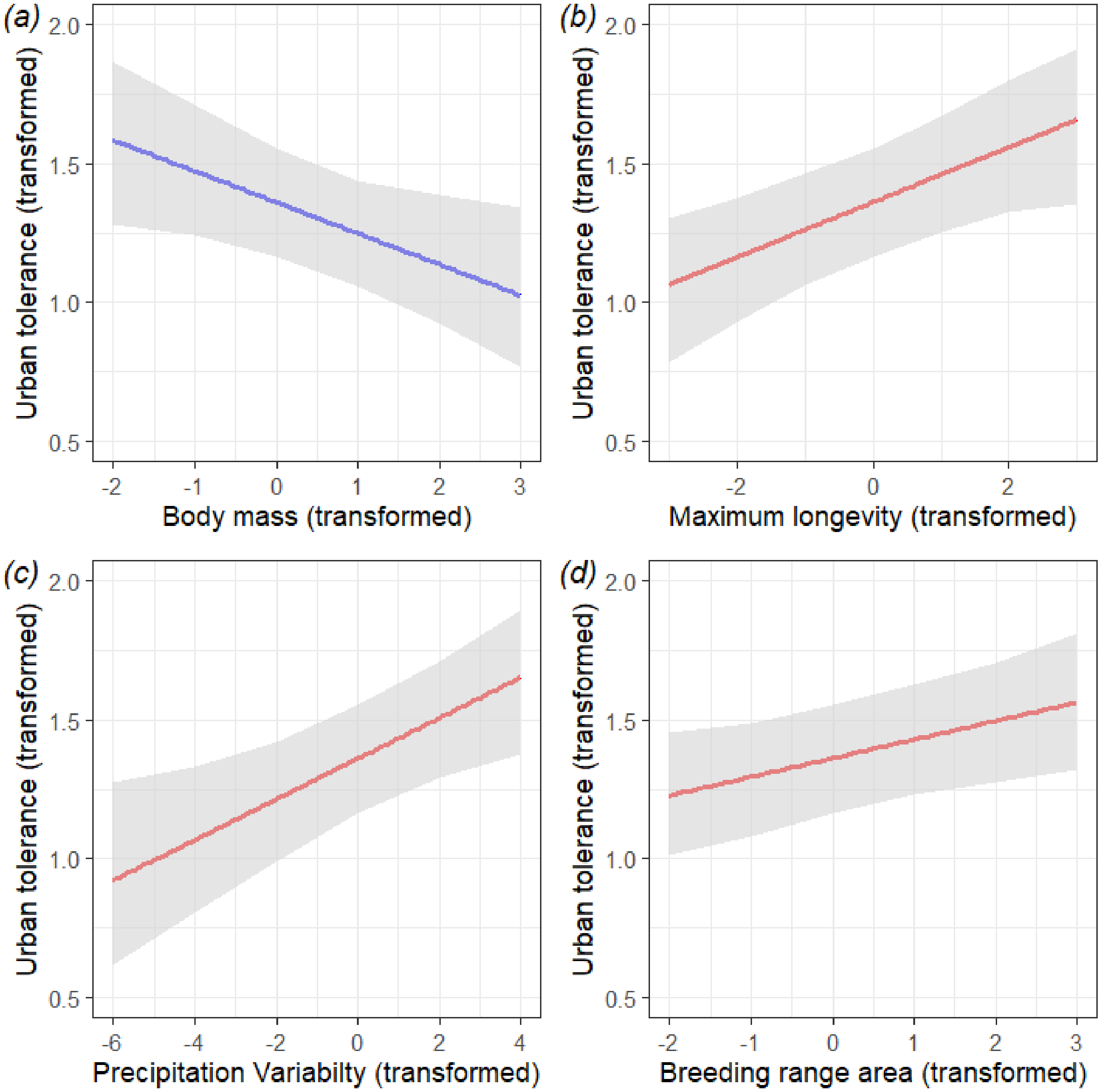

Figure S2. A Bayesian phylogenetic mixed-effects model assessing the potential influence of phylogenetic non-independence produced similar results to the main analysis (cf., Figure 1, main text). A species' urban tolerance (square-root transformed, y-axis) was predicted by its (a) body mass (log_{10} transformed and scaled), (b) maximum longevity (log_{10} transformed and scaled), (c) precipitation variability (square-root transformed and scaled), and (d) breeding range area (square-root transformed and scaled). Lines in plots illustrate model predicted relationships, with shaded regions representing 95% Highest Posterior Density (HPD) intervals, and solid lines representing model-predicted values (estimated marginal means across categorical predictor levels, with other continuous predictors held at their mean. The type of relationship is denoted by colour: red = positive, blue = negative. All plots illustrate relationships that control for all other effects retained in the model. See main text and electronic supplementary material S1, Tables S2 and S3 for full details.

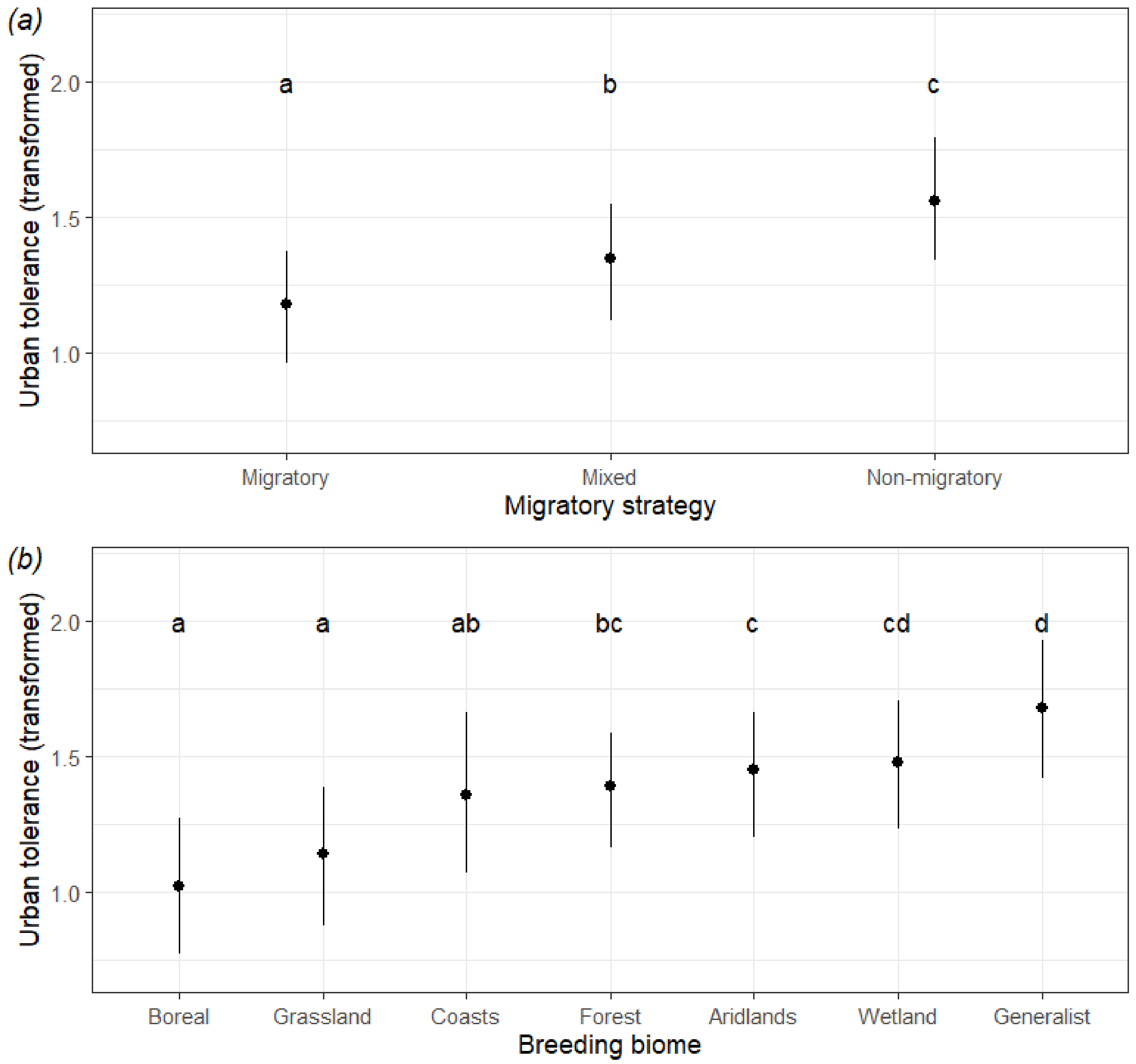

Figure S3. A Bayesian phylogenetic mixed-effects model assessing the potential influence of phylogenetic non-independence produced some varying results to the main analysis (cf., Figure 1, main text). A species' urban tolerance (square-root transformed, y-axis) was predicted by its (a) migratory strategy in both analyses, but in the main analysis urban tolerance estimates of mixed-migratory and non-migratory species did not differ (whereas they do in this analysis). Similar to the main analysis, (b) generalist species had higher urban tolerance than boreal and grassland species. Points illustrate posterior median estimates and error bars show 95% highest posterior density (HPD) interval limits. Estimates sharing a letter have pairwise contrasts with 95% HPD intervals that include zero, indicating no evidence of a difference between groups. All plots illustrate relationships that control for all other effects retained in the model. See main text and electronic supplementary material S1, Tables S2 and S3 for full details.

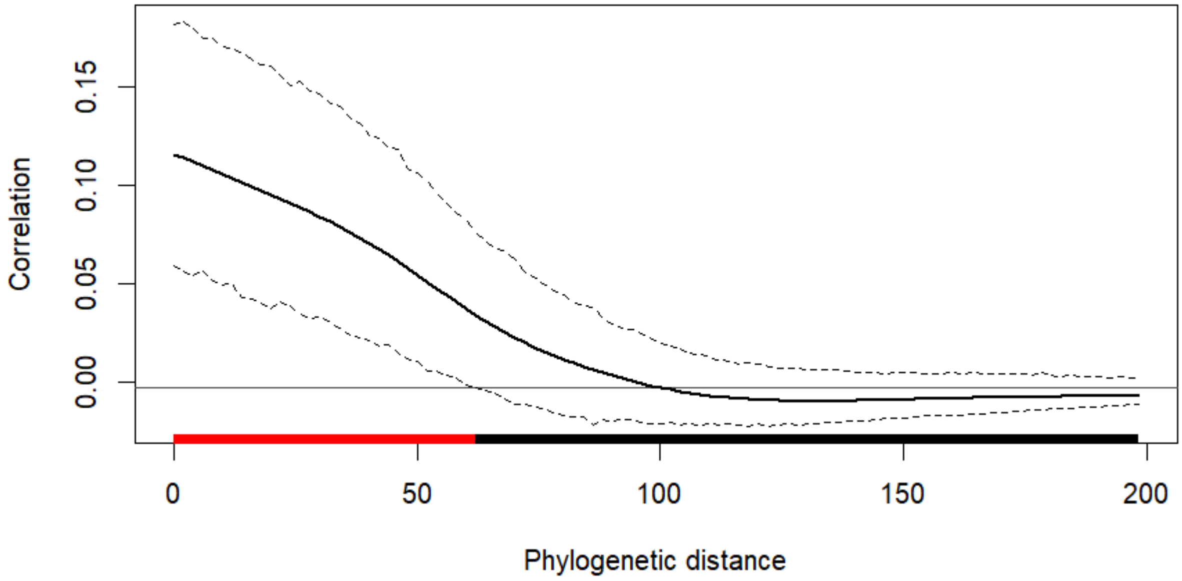

Figure S4. Evidence of a phylogenetic signal in urban tolerance score among closely-related North American birds. Plot shows an estimate of phylogenetic signal (Moran's I index of autocorrelation, y-axis) in urban tolerance estimates across phylogenetic distance (patristic distance, x-axis) for 431 North American bird species. The color of the thick bar along the x-axis illustrates the type of autocorrelation, with red denoting a positive autocorrelation ($p < 0.05$) and black a nonsignificant autocorrelation. The solid black curve represents the mean estimated Moran's I and the dashed lines illustrate the upper and lower 95% confidence interval. The horizontal line denotes the expected Moran's I value of the null hypothesis (i.e., there is no phylogenetic autocorrelation for urban tolerance).
