# Supplementary material for: Ecological and life history traits predict urban tolerance in North American birds
Source: R Soc Open Sci. 2026 Oct 14;13(10):260953. doi: 10.1098/rsos.260953 (PMC13679050; doi:10.1098/rsos.260953)
Supplement: Supplementary file 1 [file rsos.260953.zip › rsos.260953/rsos260953_si_002.pdf]

Electronic Supplementary Material 2

Ecological and life history traits predict urban tolerance in North American birds

R.E. Fanelli, P.R. Martin, and F. Bonier

Table S1. A model selection table of the top models and null model identified through comparison of all recombinant versions of a global model[1] to explain variation in urban tolerance in 431 North American bird species, with models arranged by increasing Akaike Information Criterion (AICc[2]). Categorical fixed effects retained in a model are denoted as "+".

Model Rank	Intercept	Breeding biome	Migratory status	Primary diet	Range size	Maximum longevity	Body mass	Precipitation variability	Reproductive output	Temperature variability	DF	logLik[3]	AICc	ΔAICc[4]	ω^{i}[5]
1	1.34	+	+	NA	0.05	0.09	-0.12	0.11	NA	NA	21.00	-212.42	469.11	0.00	0.24
2	1.28	+	+	+	0.05	0.09	-0.10	0.11	NA	NA	25.00	-208.88	470.97	1.87	0.09
3	1.32	+	+	NA	NA	0.10	-0.13	0.12	NA	NA	20.00	-214.47	471.00	1.89	0.09
NULL	1.39	NA	NA	NA	NA	NA	NA	NA	NA	NA	9.00	-299.72	617.86	148.75	0.00

[1] Global model: urban tolerance ~ migratory status + breeding biome + primary diet + reproductive output + temperature variability + precipitation variability + body mass + breeding range area (size) + maximum longevity. Continuous factors were transformed and scaled to facilitate comparisons (see main text for details). Taxonomic family was included as a random effect and a term was included to model the variance across levels of breeding biome to account for heteroscedasticity.

[2] AICc: Akaike's Information Criterion corrected for small sample size

[3] log-likelihood: Maximum likelihood estimate

[4] ΔAICc: the difference in AICc from the top-ranked model

[5] ω^{i}: Akaike model weights represent the relative likelihood of the model

Table S2. Summary of the top-ranked linear mixed-effects model[1] explaining variation in urban tolerance in 431 North American bird species.

Parameter	*β*±SE	*t*-value[2]	*p*-value
intercept[3]	1.334 ± 0.081	16.496	<0.001
migration strategy (mixed)	0.155 ± 0.045	3.416	<0.001
migration strategy (non-migratory)	0.286 ± 0.070	4.114	<0.001
breeding biome (boreal forest)	-0.475 ± 0.090	-5.256	<0.001
breeding biome (coasts)	-0.144 ± 0.151	-0.956	0.340
breeding biome (forests)	-0.097 ± 0.079	-1.232	0.219
breeding biome (generalist)	0.192 ± 0.096	2.000	0.046
breeding biome (grassland)	-0.355 ± 0.095	-3.727	<0.001
breeding biome (wetland)	0.003 ± 0.104	0.028	0.978
breeding range size	0.054 ± 0.025	2.182	0.030
precipitation variability	0.107 ± 0.024	4.456	<0.001
body mass	-0.118 ± 0.035	-3.334	<0.001
maximum longevity	0.0901 ± 0.028	3.211	<0.001

[1] Top-ranked model was: urban tolerance ~ migration strategy + breeding biome + breeding range size + precipitation variability + body mass + maximum longevity. Taxonomic family was included as a random effect, and a term was included to model variance structure across levels of breeding biome. See main text for details of data transformations.

[2] Degrees of freedom for all tests = 359.

[3] Intercept values for categorical predictors: migration strategy = migratory, breeding biome = aridlands. See Table S3 for pairwise comparisons of urban tolerance across all levels of these two categorical fixed effects. All continuous predictors were scaled prior to analysis to facilitate comparison of effects.

Table S3. Pairwise *post-hoc* contrasts of estimated marginal mean urban tolerance values for 431 bird species across levels of migration strategy and primary breeding biome, which were both retained as fixed effects in the top-ranked model[1]. Contrasts with *p*-values <0.05 are highlighted in bold.

Contrast	*Estimate ±SE*	*t*-value[2]	*p*-value[3]
MIGRATION STRATEGY			
**Migratory – Mixed**	**-0.155 ± 0.046**	**-3.416**	**0.002**
**Migratory – Non-migratory**	**-0.286 ± 0.070**	**-4.114**	**0.002**
Mixed – Non-migratory	-0.131 ± 0.067	-1.956	0.125
BREEDING BIOME			
Boreal – Grasslands	-0.120 ± 0.086	-1.395	0.804
Boreal – Coasts	-0.331 ± 0.153	-2.168	0.316
**Boreal – Forests**	**-0.378 ± 0.060**	**-6.293**	**<0.001**
**Boreal – Aridlands**	**-0.475 ± 0.090**	**-5.256**	**<0.001**
**Boreal – Wetlands**	**-0.478± ±.094**	**-5.078**	**<0.001**
**Boreal – Generalists**	**-0.667 ± 0.079**	**-8.411**	**<0.001**
Grasslands – Coasts	-0.211± ±.149	-1.415	0.794
**Grasslands – Forests**	**-0.258 ± 0.076**	**-3.392**	**0.014**
**Grasslands – Aridlands**	**-0.355 ± 0.095**	**-3.727**	**0.004**
**Grasslands – Wetlands**	**-0.358 ± 0.094**	**-3.797**	**0.003**
**Grasslands – Generalists**	**-0.547 ± 0.088**	**-6.252**	**<0.001**
Coasts – Forests	-0.047 ± 0.142	-0.329	0.999
Coasts – Aridlands	-0.144 ± 0.151	-0.956	0.963
Coasts – Wetlands	-0.147 ± 0.136	-1.079	0.934
Coasts – Generalists	-0.336 ± 0.150	-2.240	0.277
Forests – Aridlands	-0.097 ± 0.079	-1.232	0.881
Forests – Wetlands	-0.100 ± 0.084	-1.198	0.895
**Forests – Generalists**	**-0.289 ± 0.067**	**-4.297**	**<0.001**
Aridlands – Wetlands	-0.003 ± 0.104	-0.028	1.000
Aridlands – Generalists	-0.192 ± 0.096	-2.001	0.416
Wetlands – Generalists	-0.189 ± 0.087	-2.175	0.312

[1] Top-ranked model was: urban tolerance ~ migration strategy + breeding biome + breeding range size + precipitation variability + body mass + maximum longevity. Taxonomic family was included as a random effect, and a term was included to model variance structure across levels of breeding biome. See main text for details of data transformations.

[2] Degrees of freedom for all tests = 359.

[3] Contrasts incorporate a Tukey adjustment for multiple tests and control for other fixed effects retained in the model

Table S4. Summary of a Bayesian phylogenetic mixed-effects model in a supplemental analysis[1] explaining variation in urban tolerance in 431 North American bird species.

Parameter	β [95% CI]	N_{eff}	p-value
intercept[2]	1.271 [1.024 – 1.505]	3000	<0.001
migration strategy (mixed)	0.169 [0.068 – 0.261]	3344	0.001
migration strategy (non-migratory)	0.385 [0.248 – 0.533]	2819	<0.001
breeding biome (boreal forest)	-0.426 [-0.627 – -0.205]	2963	0.001
breeding biome (coasts)	-0.088 [-0.348 – 0.160]	300	0.495
breeding biome (forests)	-0.062 [-0.208 – 0.080]	3000	0.394
breeding biome (generalist)	0.218 [0.013 – 0.431]	3000	0.044
breeding biome (grassland)	-0.311 [-0.514 – -0.118]	3000	0.003
breeding biome (wetland)	0.023 [-0.191 – 0.234]	3000	0.815
breeding range size	0.069 [0.015 – 0.121]	2816	0.009
precipitation variability	0.07 [0.027 – 0.114]	3000	0.001
body mass	-0.108 [-0.199– -0.020]	2432	0.029
maximum longevity	0.100 [0.041 – 0.165]	3000	0.003

[1] This analysis is a supplement to the main analysis of the same sample of 431 species (summarized in Table S2) using a linear mixed-effects model that did not include phylogeny as a parameter, but included all of the fixed effects shown here. See main text for details of data transformations, as well as model priors and specifications.

[2] Intercept values for categorical predictors: migration strategy = migratory, breeding biome = aridlands.

Table S5. Pairwise *post-hoc* contrasts of estimated marginal mean urban tolerance values for 431 bird species across levels of migration strategy and primary breeding biome, which were both included in a Bayesian phylogenetic mixed-effects model supplemental analysis[1] explaining variation in urban tolerance in 431 North American bird species. Values represent posterior median estimates of pairwise contrasts with 95% highest posterior density (HPD) interval limits. Contrasts with 95% HPD intervals that exclude zero are highlighted in bold.

Contrast	*Posterior median estimate*	*95% HPD interval*
MIGRATION STRATEGY		
**Migratory – Mixed**	**-0.168**	**-0.261 – -0.068**
**Migratory – Non-migratory**	**-0.384**	**-0.533 – -0.248**
**Mixed – Non-migratory**	**-0.215**	**-0.354 – -0.086**
BREEDING BIOME		
Boreal – Grasslands	-0.118	-0.339 – 0.102
**Boreal – Coasts**	**-0.342**	**-0.639 – -0.052**
**Boreal – Forests**	**-0.365**	**-0.273 – -0.222**
**Boreal – Aridlands**	**-0.428**	**-0.627 – -0.205**
**Boreal – Wetlands**	**-0.451**	**-0.684 – -0.209**
**Boreal – Generalists**	**-0.647**	**-0.877 – -0.427**
Grasslands – Coasts	-0.222	-0.495 – 0.042
**Grasslands – Forests**	**-0.249**	**-0.416** – -0.**078**
**Grasslands – Aridlands**	**-0.310**	**-0.515 – -0.118**
**Grasslands – Wetlands**	**-0.335**	**-0.552 – -0.114**
**Grasslands – Generalists**	**-0.529**	**-0.737 – -0.308**
Coasts – Forests	-0.027	-0.273 – 0.222
Coasts – Aridlands	-0.090	-0.348 – 0.160
Coasts – Wetlands	-0.111	-0.328 – 0.101
**Coasts – Generalists**	**-0.306**	**-0.598 – -0.027**
Forests – Aridlands	-0.063	-0.208 – 0.080
Forests – Wetlands	-0.085	-0.272 – 0.097
**Forests – Generalists**	**-0.282**	**-0.454 – -0.114**
Aridlands – Wetlands	-0.025	-0.234 – 0.191
**Aridlands – Generalists**	**-0.218**	**-0.431 – -0.013**
Wetlands – Generalists	-0.195	-0.394 – 0.038

[1] Bayesian model was: urban tolerance ~ migration strategy + breeding biome + breeding range size + precipitation variability + body mass + maximum longevity. Taxonomic family was included as a random effect, and a term was included to model variance structure across levels of breeding biome. This analysis is a supplement to the main analysis of the same sample of 431 species (summarized in Table S2) using a linear mixed-effects model that did not include phylogeny as a parameter, but included all of the fixed effects shown here. See main text for details of data transformations, as well as model priors and specifications.
